# The AAA-ATPase VPS4 Regulates Extracellular Secretion and Lysosomal Targeting of α-Synuclein

**DOI:** 10.1371/journal.pone.0029460

**Published:** 2011-12-22

**Authors:** Takafumi Hasegawa, Masatoshi Konno, Toru Baba, Naoto Sugeno, Akio Kikuchi, Michiko Kobayashi, Emiko Miura, Nobuyuki Tanaka, Keiichi Tamai, Katsutoshi Furukawa, Hiroyuki Arai, Fumiaki Mori, Koichi Wakabayashi, Masashi Aoki, Yasuto Itoyama, Atsushi Takeda

**Affiliations:** 1 Division of Neurology, Department of Neuroscience & Sensory Organs, Tohoku University Graduate School of Medicine, Sendai, Miyagi, Japan; 2 Division of Cancer Biology and Therapeutics, Miyagi Cancer Center Research Institute, Natori, Miyagi, Japan; 3 Department of Geriatrics and Gerontology, Institute of Development, Aging and Cancer, Tohoku University, Sendai, Miyagi, Japan; 4 Department of Neuropathology, Institute of Brain Science, Hirosaki University School of Medicine, Hirosaki, Aomori, Japan; 5 National Center Hospital for Mental, Nervous, and Muscular Disorders, National Center of Neurology and Psychiatry, Kodaira, Tokyo, Japan; University of Maryland School of Pharmacy, United States of America

## Abstract

Many neurodegenerative diseases share a common pathological feature: the deposition of amyloid-like fibrils composed of misfolded proteins. Emerging evidence suggests that these proteins may spread from cell-to-cell and encourage the propagation of neurodegeneration in a prion-like manner. Here, we demonstrated that α-synuclein (αSYN), a principal culprit for Lewy pathology in Parkinson's disease (PD), was present in endosomal compartments and detectably secreted into the extracellular milieu. Unlike prion protein, extracellular αSYN was mainly recovered in the supernatant fraction rather than in exosome-containing pellets from the neuronal culture medium and cerebrospinal fluid. Surprisingly, impaired biogenesis of multivesicular body (MVB), an organelle from which exosomes are derived, by dominant-negative mutant vacuolar protein sorting 4 (VPS4) not only interfered with lysosomal targeting of αSYN but facilitated αSYN secretion. The hypersecretion of αSYN in VPS4-defective cells was efficiently restored by the functional disruption of recycling endosome regulator Rab11a. Furthermore, both brainstem and cortical Lewy bodies in PD were found to be immunoreactive for VPS4. Thus, VPS4, a master regulator of MVB sorting, may serve as a determinant of lysosomal targeting or extracellular secretion of αSYN and thereby contribute to the intercellular propagation of Lewy pathology in PD.

## Introduction

Although the pathophysiology of Parkinson's disease (PD) is still a topic of debate, the current consensus is that the cytoplasmic accumulation of fibrillar α-synuclein (αSYN) in the affected brain lesions is a hallmark of the initiation and progression of the disease [1], [2], [3], [4], [5]. In human brain, αSYN is enriched in presynaptic nerve terminals and is mainly detected both in cytosolic and synaptosomal fractions [6], [7]. On the other hand, both monomeric and oligomeric αSYN has been found in the neuronal culture medium as well as in body fluids such as plasma and cerebrospinal fluid (CSF) [8], [9], [10], [11]. The existence of extracellular αSYN is also supported by the fact that the hydrophobic core region of αSYN, termed NAC (non-amyloid-β component), is observed in the extracellular senile plaques of Alzheimer's disease (AD) [12]. The biochemical influence of extracellular αSYN is not understood yet, but *in vitro* generated soluble αSYN oligomers can induce transmembrane seeding of αSYN aggregation and eventually cause neuronal cell death [13]. The intercellular transmission of αSYN is also verified by co-culture experiments and *in vivo* animal models showing that αSYN aggregates released from neuronal cells can be transferred to neighboring cells and form intracellular inclusions [14], [15], [16], [17]. Moreover, it has been shown that αSYN-containing conditioned medium not only induced neuronal death, but also triggered inflammatory responses in astroglial cells [15]. Finally, the *in vivo* cell-to-cell propagation of pathogenic protein was strongly supported by recent observations showing that αSYN-positive, Lewy body-like cytoplasmic inclusions were found in fetal mesencephalic neurons that were transplanted into the brain of PD patients more than a decade ago [18], [19], [20]. This scenario is immensely attractive as an acceptable explanation for the clinically observed progression of neurodegenerative diseases as well as the stereotypic spread of Lewy pathology suggested by Braak and his colleagues [21].

The cellular and molecular mechanisms by which intercellular transmission of infectious prions occurs are still enigmatic. Nevertheless, several reports revealed that both normal cellular prion protein (PrPc) and the abnormally folded pathogenic form (PrPsc) were associated with nanovesicles called ‘exosomes’ released from non-neuronal and neuronal cells [22], [23], [24], [25]. Once released from a cell it is proposed that exosomes could fuse with the plasma membrane of neighboring cells, transferring exosomal molecules from one cell to another. Vesicles with the hallmarks of exosomes have been detected in a large variety of biological fluids including saliva, serum/blood, urine and CSF [26]. Very recently, it was shown that part of the cell-produced αSYN can be secreted via an exosomal, calcium-dependent mechanism and that the exosome-containing conditioned medium from αSYN-expressing cells caused the cell death of recipient neuronal cells [27]. Another piece of evidence showed that lysosomal dysfunction led to an increase in the release of αSYN in exosomes and a concomitant increase in αSYN transmission to recipient cells [28]. These findings raise the possibility that methods to prevent pathogenic protein trafficking and propagation could be designed from insights concerning the mechanisms involved in exosome biogenesis.

Multivesicular bodies (MVBs), the endocytic organelles from which exosomes are derived, are generated from the invagination of the limiting membrane into the luminal space [29], [30], [31]. MVBs are involved in the sequestration of proteins that are condemned to lysosomal degradation. An alternative destination of MVBs is their exocytic fusion with the plasma membrane leading to the release of intraluminal vesicles (ILVs; i.e., exosome) into the extracellular environment. Mechanistically, the sorting of cargo proteins into ILVs from MVBs is a tightly regulated process that depends on a functional complex called ESCRT (Endosomal Sorting Complex Required for Transport) [32], [33], [34]. This highly conserved machinery consists of three distinct but cooperative functions: first, it recognizes ubiquitylated cargo protein; second, it promotes membrane deformation, facilitating the cargo to be sorted into endosomal invaginations; third, it catalyzes the final perimeter membrane scission of the endosomal invagination, which forms ILVs containing the sorted cargo [35]. During these processes, AAA (ATPases Associated with diverse cellular Activities)-ATPase VPS4 (Vacuolar Protein Sorting 4) is required for the final ESCRT-disassembly, which completes the membrane abscission and is thus indispensable for MVB biogenesis [36]. Functional VPS4 is composed of two parallel hexameric rings made of VPS4A and B. It is known that VPS4 paralogues are differentially expressed in different organs, e.g., the expression of VPS4A is higher than that of VPS4B in mouse brain. [37].

We now report that, in contrast to PrP, extracellular αSYN was mainly detected in the supernatant fraction rather than in exosome-containing pellets from neuronal culture medium (CM) and CSF. Furthermore, perturbation of MVB-exosome genesis by dominant negative (DN) VPS4A unexpectedly increased extracellular αSYN concomitant with decreased lysosomal targeting of αSYN. The aberrant secretion of αSYN induced by VPS4 malfunction was effectively restored by the functional disruption of recycling endosome regulator Rab11a. Our results uncover a novel functional role of the MVB sorting pathway in the extracellular secretion as well as lysosomal targeting of αSYN.

## Results

### α-Synuclein Is Present in Endosomal Compartments

**Figure 1:**
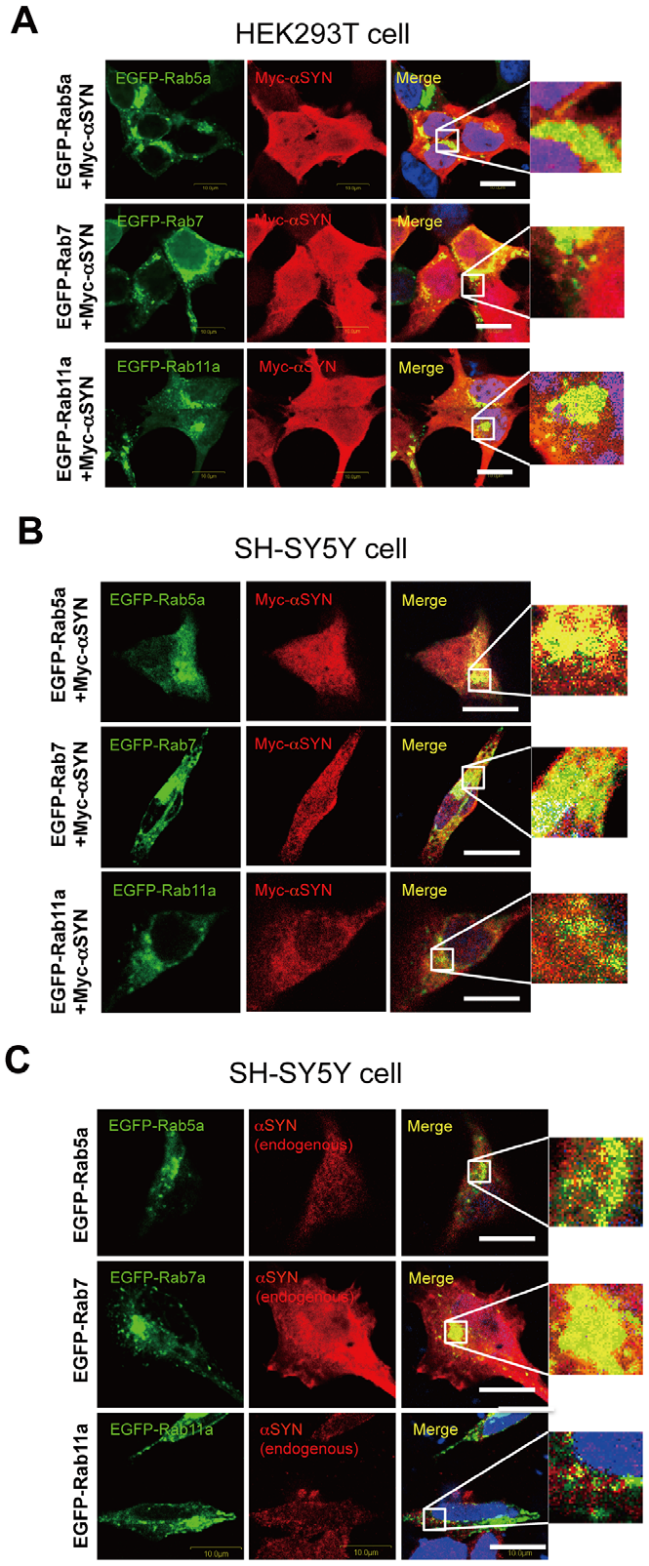
α-synuclein is present in endosomal compartments. Colocalization experiments of Myc-tagged αSYN (red) with endosome-associated EGFP-tagged Rab proteins (green) in HEK293T cell (*A*) and SH-SY5Y dopaminergic neuronal cells (*B*). The subcellular distribution of endogenous αSYN was also examined in SH-SY5Y cells expressing EGFP-tagged Rab proteins (*C*). Cells were fixed 48 hours post-transfection and were subjected to immunofluorescent analysis. In both cell lines, exogenously expressed αSYN was detected not only throughout the cytosol but also in punctate endosomal structures that were positive for EGFP-Rab5a (early endosome marker), Rab7 (late endosome marker), and Rab11a (recycling endosome marker), respectively. Nuclei were conterstained with TO-PRO3 iodide (pseudocolored blue). The inset picture is a magnified picture of the square area. Immunostaining was performed three times and the experiment three times with the same results. Size bar: 10 µm.

In eukaryotic cells, endosomes comprise three different compartments: early endosomes, late endosomes, and recycling endosomes. They are not only distinguished by morphology, differential density, and internal pH, but also by the specific localization of Rab GTPases [38], [39]. To determine whether αSYN is actually localized in the endosomal compartment in cultured cells, we transiently transfected Myc-αSYN-expressing HEK293T and human neuronal SH-SY5Y cells together with EGFP-tagged Rab GTPase Rab5a, Rab7, and Rab11a, which are indispensable effectors/constituents of early endosomes, late endosomes, and recycling endosomes, respectively [39]. The reason why we used HEK293T cells is that they are very easy to transfect and the level of protein expression is very high. As shown in Fig. 1A and B, exogenously expressed αSYN in both cells was clearly detected not only throughout the cytosol but also in punctate endosomal structures, which were positive for EGFP-Rab5a, Rab7, and Rab11a. The partial co-localization of endogenous αSYN with endosomal Rab proteins was also observed in human neuronal SH-SY5Y cells (Fig. 1C). The patterns of distribution of EGFP-tagged Rab family proteins were quite distinct from those of the EGFP-expressing cells, showing diffuse cytoplasmic signals throughout the cytosol (data not shown).

### α-Synuclein Is Detected in Supernatant But Not in Exosome-Containing Pellet from Neuronal Culture Medium and CSF

**Figure 2:**
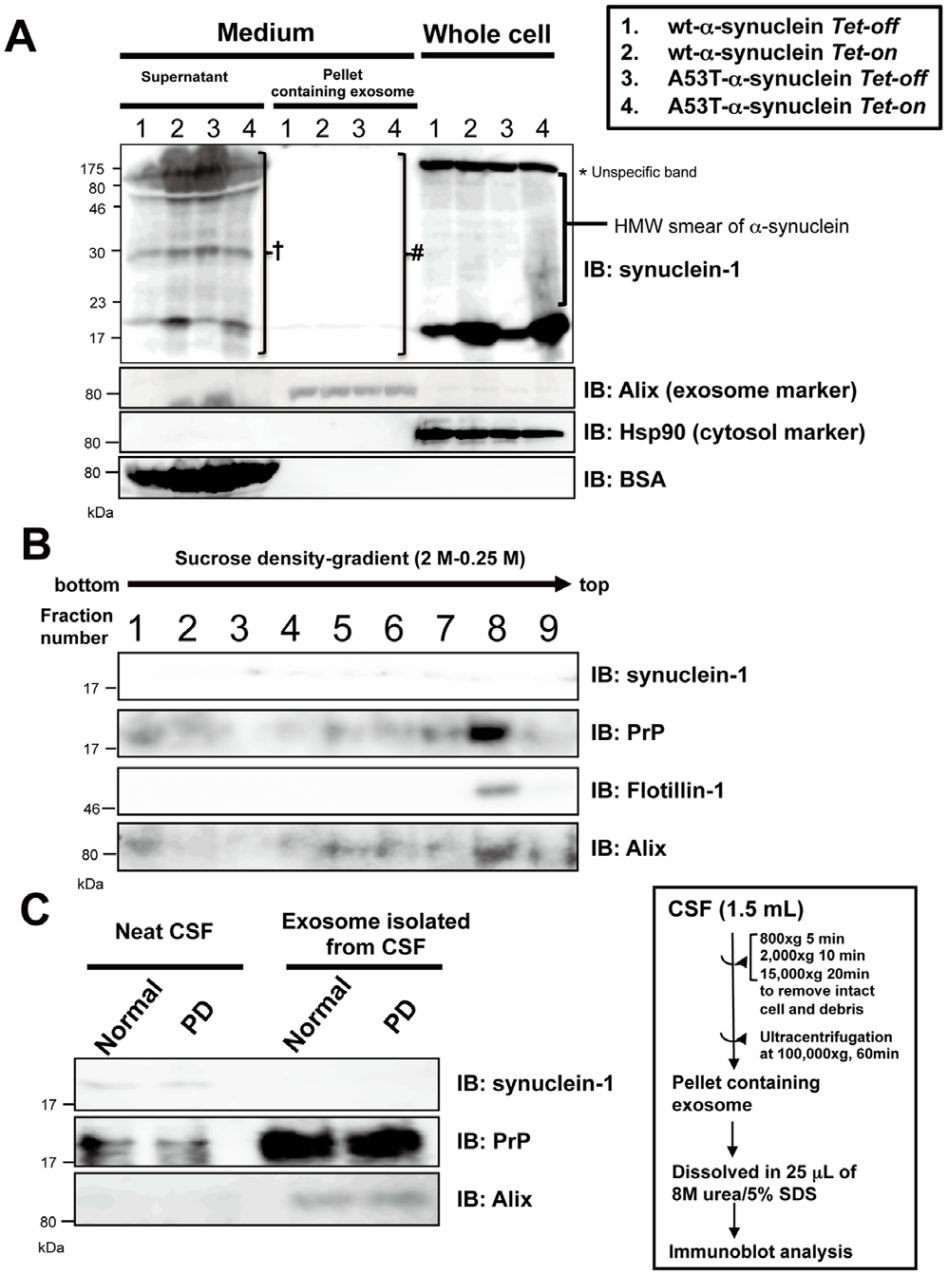
α-synuclein is detected in supernatant but not in the exosome-containing pellets from neuronal culture medium and CSF by standard immunoblot analysis. *A*. wt and A53T mutant αSYN were inducibly expressed in SH-SY5Y cells for 48 hours. Culture medium as well as whole cell lysates (50 µg protein per lane) were subjected to Western blot analysis. The collected media were further separated into the supernatant and exosome-containing pellets before loading onto gels. Alix, Hsp90, and BSA were used as markers for exosome, cytosol, and culture medium, respectively. In the neuronal culture medium, both monomeric/oligomeric wt and mutant αSYN were recovered in the supernatant (*dagger*) rather than exosome-containing pellets (*hash*). *Asterisk* indicates unspecific band. *B*. The resuspended exosome-containing pellets from the culture medium were further separated by sucrose-density gradient followed by Western blot analysis. Immunoblot probed with synuclein-1 anti-αSYN, anti-PrP Abs and the successful separation of exosome was confirmed by exosomal markers, Flotillin-1 and Alix. As shown in the blot, PrP migrated near the top of the density gradient (fraction #8) with concomitant enrichment of exosome-associated proteins. By contrast, no exosomal enrichment was observed with αSYN. *C*. CSF (1.5 mL) from 5 PD patients together with age-matched controls was pooled and exosome-containing pellets were isolated by successive centrifugation indicated. Equal concentrations (50 µg per lane) of total CSF samples were loaded alongside CSF-derived exosomes and then probed with anti-αSYN and PrP antibodies. PrP detected in CSF-derived exosomes was enriched compared to neat CSF. αSYN was weakly but specifically detected in neat CSF, whereas no αSYN-positive signal could be detected in CSF-derived exosomes. No significant difference was observed in the expression levels of CSF αSYN between PD patients and normal controls. Representative Western blots from three independent experiments are presented.

To investigate whether αSYN is released in association with exosomes into the extracellular milieu, we induced wt and A53T mutant αSYN expression in SH-SY5Y cells and examined the CM as well as whole cell lysates for the presence of αSYN (Fig. 2A). The collected medium was further separated into the supernatant and an exosome-containing pellet, and the successful separation was verified using the exosome marker Alix. After induction, αSYN monomer and high molecular weight (HMW) αSYN smear were significantly increased in the cell lysates. A53T mutant αSYN had a high propensity to form HMW smear, as previously reported [2], [40]. Following the induction, wt and, to a lesser extent A53T mutant αSYN, in the supernatant of CM were easily detected and dramatically increased. However, the expression levels of αSYN in the exosome-containing pellet were very weak and unchanged even after the induction. Thus, it is supposed that the majority of secreted αSYN in CM is not concealed in exosome vesicles, but released directly into the supernatant. We confirmed that the presence of αSYN in CM was not attributable to disruption of the cellular membrane since Hsp90, the most abundantly expressed protein in the cytosol of eukaryotic cells, could not be detected in the samples prepared from CM. To confirm the extracellularαSYN localization in more detail, the resuspended exosome-containing 100,000×*g* pellets obtained from CM were further analyzed by floatation in a continuous sucrose-density gradient (Fig. 2B). As expected, Western blot analysis of the separated fractions revealed that PrP migrated near the top of the density gradient with concomitant enrichment of the exosome-associated proteins, Alix and Flottilin-1. By contrast, only trace amounts of αSYN were broadly detected in the sucrose gradient and no exosomal enrichment was observed. The separation appeared to be successful since exosomes have been reported to float on sucrose gradients at density ranges depending on the cell type [41].

PrP can be detected in several biological fluids such as blood, lymph, and CSF, which are confirmed to be sources of prion infectivity [42]. Furthermore, exosomes isolated from ovine CSF were an efficient means of enriching PrPc and PrPsc suitable for detection using Western blot analysis [41]. While detectable amounts of αSYN have also been identified in human blood plasma and CSF [8], [9], [10], [11], it has not yet been determined whether αSYN is enriched in exosomes derived from CSF. In an attempt to examine whether CSF-derived exosomes were enriched in αSYN relative to neat CSF, we pooled CSF samples from five different PD patients together with age-matched controls and then the exosomes were isolated by ultracentrifugation. Equal concentrations (50 µg per lane) of total CSF samples were loaded alongside CSF-derived exosomes and then probed with anti-αSYN and PrP antibodies (Fig. 2C). The amount of PrP detected in CSF-derived exosomes was enriched compared to neat CSF in which signals were only weakly observed. We confirmed that αSYN was weakly but specifically detected in neat CSF; however, we failed to detect αSYN-positive signals in CSF-derived exosomes by standard immunoblotting technique. There was no significant difference in the expression levels of CSF αSYN between PD patients and normal controls.

### Expression of DN VPS4A Leads to Increased Extracellular α-Synuclein and a Parallel Decrease in Lysosomes

**Figure 3:**
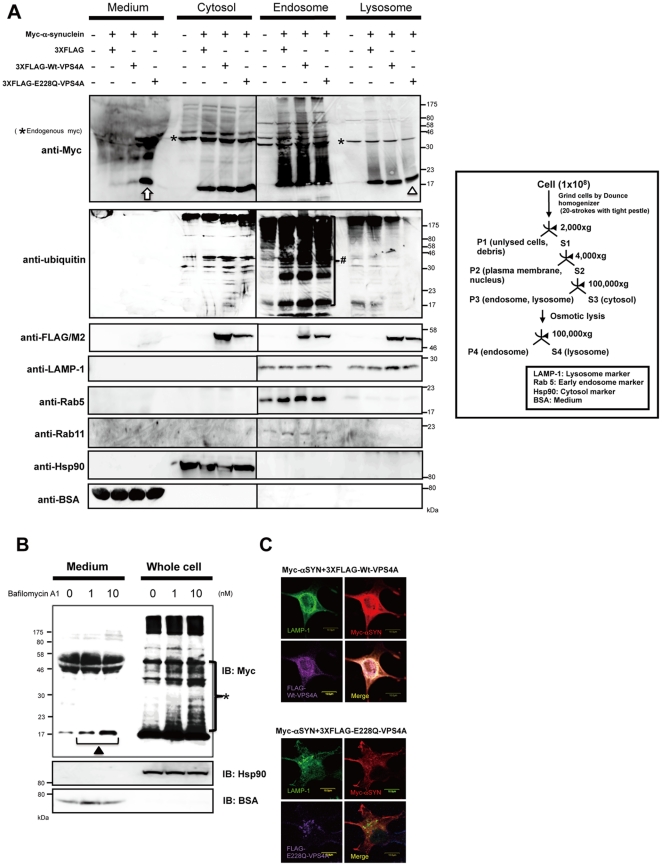
Over-expression of DN VPS4 in HEK293T cells leads to increased extracellular α-synuclein and parallel decrease in lysosome. *A*. αSYN-expressing HEK293T cells were co-transfected either with 3XFLAG-tagged wt-VPS4A or DN mutant (E228Q) VPS4A. Forty-eight hours after transfection, HEK293T cells were fractionated into the cytosol (S3), endosome (P4), and lysosome (S4). Fractionated cell lysates as well as protein extracts from the culture medium (50 µg protein per lane) were subjected to Western immunoblot analysis using anti-Myc, anti-ubiquitin, anti-FLAG/M2 Abs. Each fraction was verified by the presence of a specific marker protein: LAMP-1 (late endosome and lysosome), Rab5 (early endosome), Rab11 (recycling endosome), Hsp90 (cytosol), and BSA (culture medium). As shown in the blot, marked increase of extracellular αSYN monomer and multimers (*white arrow*) concomitant with slightly decreased lysosomal αSYN-immunopositive smear (*open triangle*) were observed by over-expression of DN VPS4A. Note that endosomal proteins including αSYN seemed to be heavily ubiquitylated (*hash*). *Asterisk* indicates endogenous myc band. *B*. Treatment with lysosomal inhibitor bafilomycin A1 (0–10 nM) for 24 hours induced the buildup of cellular αSYN oligomers (*asterisk*) in parallel with the increase of extracellular αSYN monomer (*closed triangle*). *C*. Subcellular localization of Myc-αSYN (red) in HEK293T cells expressing wt or DN VPS4A (magenta). LAMP-1 (green) was used as a marker for late endosome and lysosome. DN VPS4 distibuted as aberrant cytoplasmic punctate structures, showing a marked contrast to wt-VPS4A with diffuse perinuclear distribution. Representative Western blots from three separate experiments are shown. Scale bar: 10 µm.

**Figure 4:**
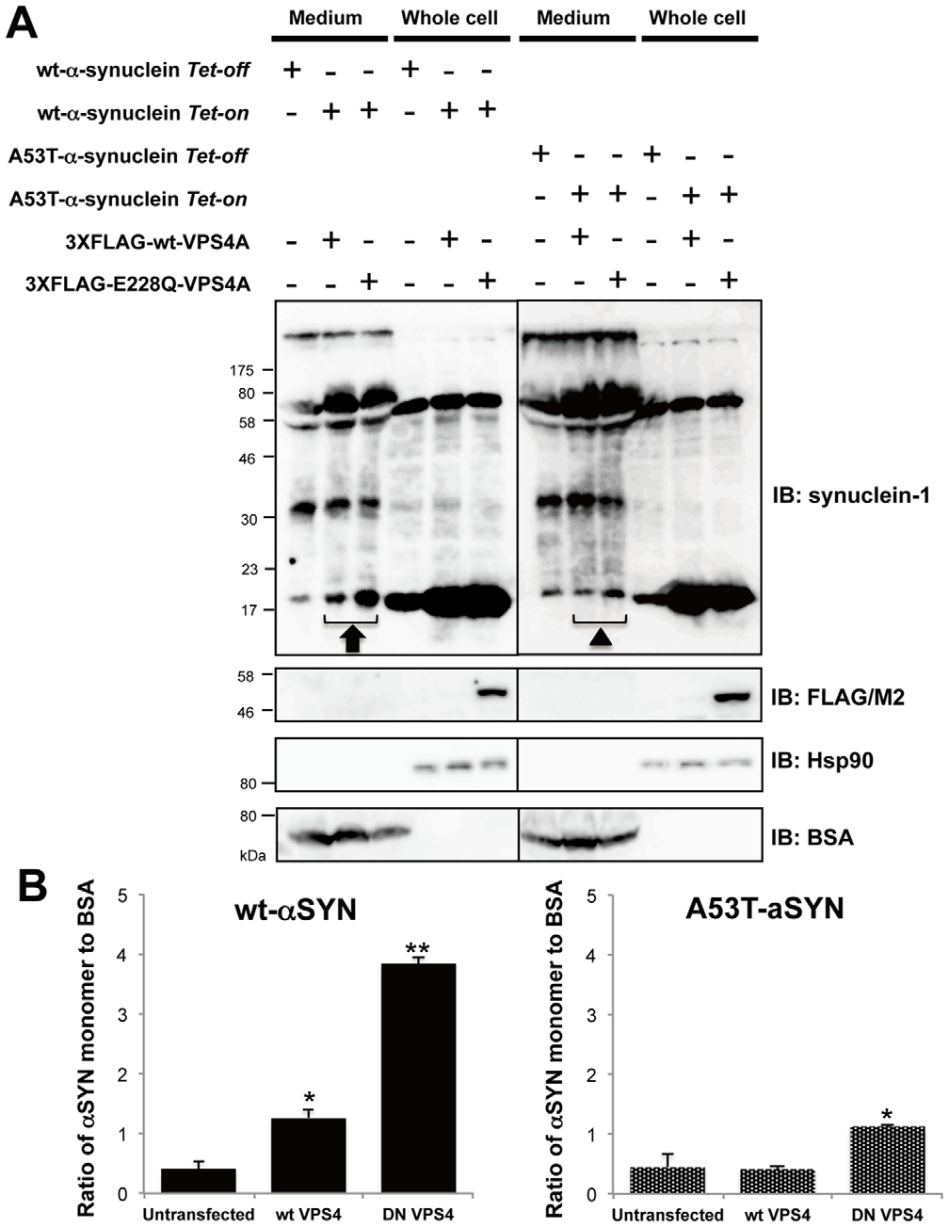
Expression of DN VPS4A increases wt as well as A53T mutant α-synuclein in SH-SY5Y neuronal cells. *A*. SH-SY5Y cells inducibly expressing wt or A53T mutant αSYN were further co-transfected with 3XFLAG-tagged wt or DN E228Q mutant VPS4A plasmids. After 48 hours of αSYN induction with doxycycline, whole cell and proteins from culture media (50 µg protein per lane) were subjected to immunoblot analysis using anti- synuclein-1 and anti-FLAG/M2 antibody. Hsp90 and BSA were used as markers for cytosol and culture medium, respectively. Increased extracellular secretion of wt and A53T mutant αSYN were observed by DN VPS4A. Note that the extracellular secretion of monomeric wt-αSYN (*black arrow*) was higher than that of A53T mutant αSYN (*closed triangle*) in mock-transfected cells as well as in DN-VPS4A engineered cells. Representative immunoblots from three independent experiments are shown. *B*. Densitometric measurement of monomeric αSYN secreted into culture media. Values indicate the ratio of αSYN monomer to BSA. Significant increase of wt as well as A53T αSYN in culture media was observed by co-expression of wt and or DN VPS4A (*p<0.05, **p<0.005).

Exosomes, by definition, correspond to the ILV of MVB, and therefore targeting a component of the ESCRT machinery could be used to interrupt protein sorting to ILV and exosome formation [22], [30]. In fact, it has been shown that disturbed ILV formation by the over-expression of DN-VPS4A induced PrPc entrapment at the limiting membrane of endosomes in rabbit epithelial Rov9 cells [43]. Thus, we hypothesized that, if αSYN secretion largely depends on exosomes as well as PrPc, functional disruption of the ESCRT components by DN-VPS4A could decrease extracellular αSYN. To prove this, αSYN-expressing HEK293T cells were co-transfected either with 3XFLAG-tagged wt-VPS4A or DN mutant (E228Q) VPS4A harbouring a single amino acid exchange in its AAA domain [44]. Forty-eight hours post-transfection, the cells were harvested and sequentially fractionated into cytosolic, endosomal, and lysosomal fractions. In parallel, proteins in cultured medium were isolated by TCA/acetone precipitation. All samples were subjected to immunoblot analysis and the relative purity of the fractions was assessed using antibodies against specific markers including LAMP-1 (lysosome), Rab5 (early endosome), Rab11 (recycling endosome), Hsp90 (cytosol), and BSA (CM), respectively. The results, shown in Fig. 3A, revealed that exogenous expression of DN-VPS4A, and a lesser extent wt-VPS4A, caused an unexpected increase of both monomeric and oligomeric αSYN in CM compared to mock (3XFLAG peptide)-transfected control. The partial increase of αSYN secretion by wt-VPS4A expression could be explained by previous data demonstrating that even wt-VPS4A was able to negatively perturb the ESCRT pathway when heavily over-expressed [45]. An intriguing finding was that the increased αSYN secretion into CM was accompanied by a slight decrease of lysosomal HMW αSYN smear (i.e., oligomers), since the autophagic-lysosomal pathway had been thought to be essential for the clearance of αSYN aggregates [46], [47], [48]. In accord with these findings, we confirmed that bafilomycin A1, a cell-permeant inhibitor of vacuolar type H+-ATPase, which plays a pivotal role in acidification and protein degradation in lysosomes, induced the buildup of cellular αSYN oligomers in parallel with the increase of its extracellular secretion in a dose-dependent manner (Fig. 3B). It is also interesting to note that the accumulated αSYN oligomers in HEK293T cells were prominent in CM, endosomal, and lysosomal compartments compared to those in the cytosol, which is in good agreement with a previous study showing that αSYN is more prone to aggregate in vesicular structures compared to the cysotol [49]. Also note that endosomal proteins including αSYN seemed to be heavily ubiquitylated compared to αSYN in other fractions. As previously reported, immunostaining revealed that exogenous expression of DN-VPS4A in HEK293T cells led to the appearance of aberrant cytoplasmic punctate structures, providing a distinct contrast to the diffuse perinuclear distribution of wt-VPS4A (Fig. 3C) [43]. We confirmed that the aberrant secretion of αSYN by DN-VPS4A expression was not a cell-type-specific phenomenon in HEK293T cells since we observed an identical result in SH-SY5Y neuronal cells, namely, wt as well as A53T mutant αSYN secretion was significantly increased by the nucleofection of wt- and DN-VPS4A (Fig. 4A). Note that the extracellular secretion of monomeric wt-αSYN was much higher than that of A53T mutant αSYN in mock-transfected cells as well as in DN-VPS4A engineered cells (Fig. 4B). Nucleofection of SH-SY5Y cells using the Nucleofector device provided a technique for introducing constructs into SH-SY5Y cells with ∼70% efficiency as estimated from the EGFP fluorescence at 48 hours post-transfection (our unpublished data).

### VPS4 is found in the core structures of Lewy bodies

**Figure 5:**
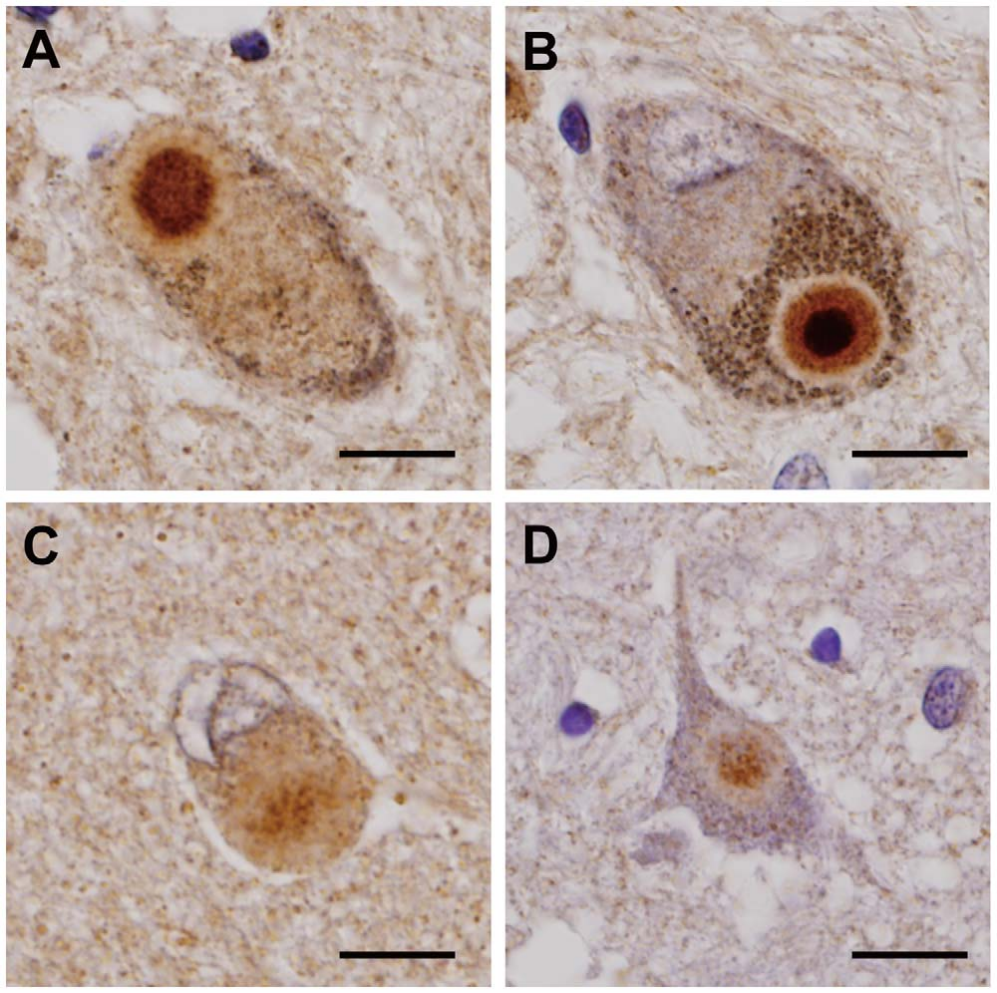
VPS4 was found to be a component of Lewy body. Paraffin embedded sections including the substantia nigra and the temporal lobes from four patients with PD with a mean age of 77.5 years and the controls with a mean age of 77.3 years were subjected to immunohistochemical analysis using anti-human VPS4 Ab. Diaminobenzidine were used to visualize the staining and nuclei were counterstained with hematoxylin. In all brain tissues from patients with PD, the core structures of Lewy bodies showed VPS4 immunoreactivity (Fig. 5). Only weak background staining was observed in control brain sections (data not shown). The percentage of VPS4-immunoreactive Lewy bodies in the substantia nigra (A and B) and the temporal lobes (C and D) of PD brains were 90% and 10%, respectively. Scale bar: 20 µm.

As shown in Fig. 3A, we found that αSYN in endosome and lysosome is more prone to aggregate than in cytosol. This result implies that endosomal/lyosomal organelles containing αSYN aggregates might be the potential source of Lewy bodies. To prove this, the substantia nigra and the temporal lobes from four patients with PD and four age-matched controls dying from known, non-neurological causes were subjected to immunohistochemical analysis using anti-human VPS4 Ab. In all brain tissues from PD patients, the core structures of Lewy bodies showed VPS4 immunoreactivity (Fig. 5), whereas only weak background staining was observed in control brain sections (data not shown). The percentage of VPS4-immunoreactive Lewy bodies in the substantia nigra (A and B) and the temporal lobes (C and D) of four PD brains are 90% and 10%, respectively.

### Increased Secretion of α-Synuclein by DN-VPS4A Is Restored by DN-Rab11a

**Figure 6:**
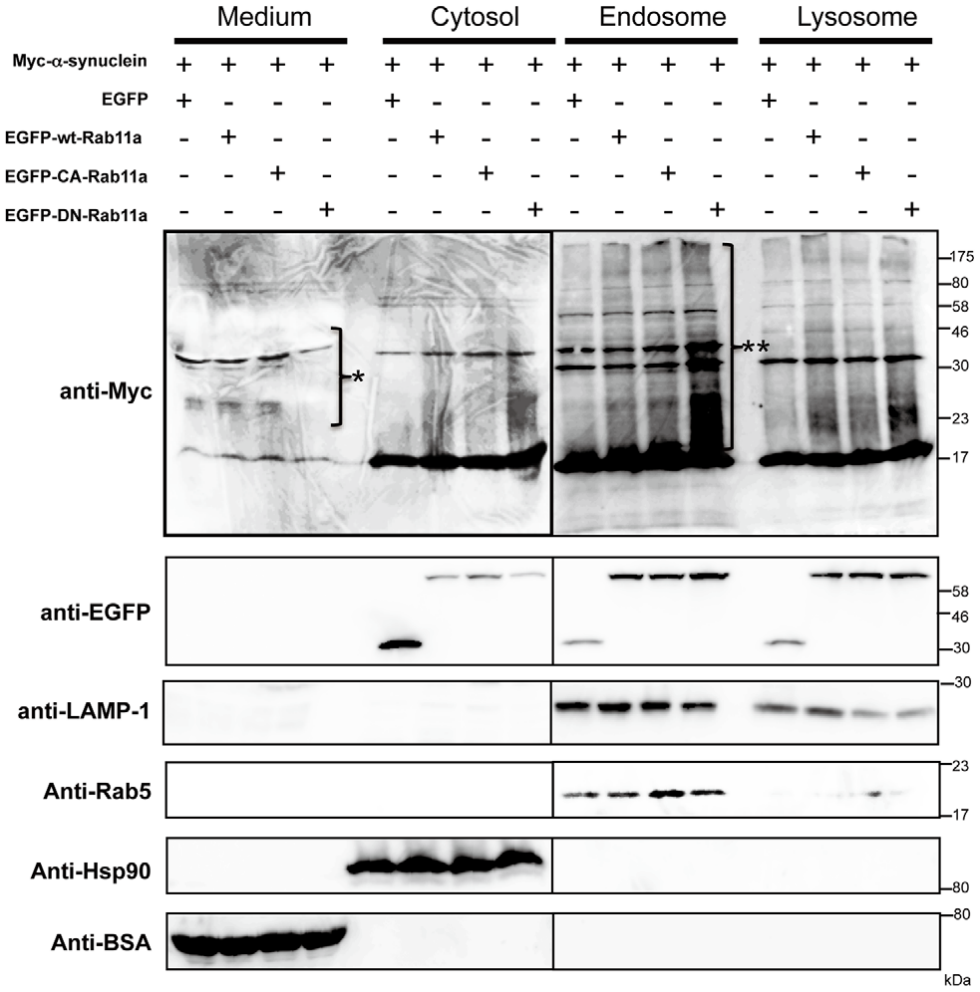
Part of the cellular α-synuclein was trafficked via a recycling endosome pathway for extracellular secretion. HEK293T cells expressing Myc-αSYN were co-transfected with mock (EGFP), EGFP-wt-Rab11a, EGFP-CA-Rab11a, or EGFP-DN-Rab11a expression plasmids. At 48 hours following transfection, the cells were harvested and fractionated into cytosol, endosome, and lysosome. Fractionated samples as well as total proteins from the culture media (50 µg per lane) were subjected to immunoblot analysis using anti-Myc, anti-EGFP Abs. A successful fraction was verified by the presence of a specific marker proteins. As shown in the blot, secretion of αSYN oligomer in culture medium was partly reduced by the over-expression of GDP-locked DN-Rab11a (*asterisk*), accompanied by the extensive retention of HMW αSYN species in the endosome (*double asterisk*). Representative blots from three separate experiments are shown.

**Figure 7:**
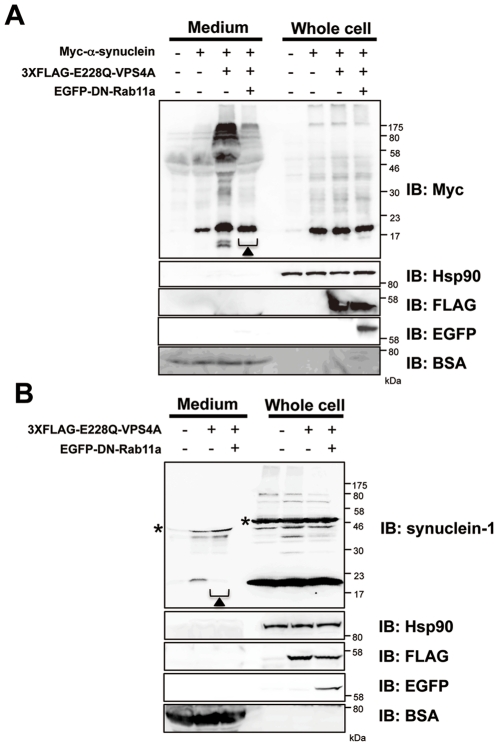
Increased secretion of α-synuclein by DN-VPS4A is restored by DN-Rab11a. GDP-locked DN-Rab11a strikingly restored the hypersecretion of αSYN triggered by the impaired MVB sorting pathway (*closed triangle*). HEK293T cells co-expressing Myc-αSYN and 3XFLAG-DN-VPS4A (*A*) and SH-SY5Y neuronal cells expressing 3XFLAG-DN-VPS4A (*B*) were further transfected with EGFP-DN-Rab11a. Forty eight hours post transfection, the cells were harvested and solubilized in RIPA buffer. Whole cell lysates as well as total proteins from culture media (50 µg per lane) were then subjected to immunoblot analysis using anti-Myc, anti-synuclein-1, anti-FLAG, and anti-EGFP Abs. Hsp90 and BSA were used as markers for the cytosol and culture medium, respectively. *Asterisk* indicates unspecific band. Representative blots from three independent experiments are presented.

It was shown that αSYN incorporated from the extracellular space was able to be resecreted out of neurons via a process modulated by recycling endosome regulator Rab11a [50]. To test the possible implication of the Rab11a-dependent recycling pathway in the secretion of αSYN *in vivo*, αSYN-expressing HEK293T cells were co-transfected with EGFP, EGFP-tagged wt-Rab11a, Q70L constitutively active (CA)-Rab11a, or S25N DN-Rab11a construct, respectively (Fig. 6). The S25N point mutation in Rab11a has been known to increase its activity for GDP, thereby locking the Rab GTPase in an inactive, non-membrane-associated state [51]. In comparison with EGFP, wt-Rab11a, and CA-Rab11a expressing cells, the cells expressing DN-Rab11a showed a slight decrease of the extracellular oligomeric αSYN in CM as well as the appearance of αSYN-immunopositive HMW smear in the endosome and, to a lesser extent, cytosolic and lysosomal fractions. This finding indicated that a part of endogenous αSYN was trafficked via a recycling endosome pathway for extracellular secretion, and the reduced recycling efficiency by DN-Rab11a expression would probably yield the aberrant retention of αSYN both in endosomes and lysosomes. Given the role of Rab11a in regulating the secretion of cellular αSYN, we speculated that the Rab11a-regulated recycling pathway could also be involved in the hypersecretion of αSYN from HEK293T cells transfected with DN-VPS4A. To test this, HEK293T cells doubly expressing αSYN and DN-VPS4A or SH-SY5Y neuronal cells expressing DN-VPS4A were further co-transfected with DN-Rab11a that lacks GTP-binding activity, then whole cell lysates as well as CM were subjected to immunoblot analysis (Fig. 7A and B, respectively). As shown in the blots, the augmented secretion of over-expressed and endogenous αSYN induced by VPS4 malfunction were effectively restored by the co-expression of GDP-locked DN-Rab11a, whereas the total cellular levels of αSYN remained unchanged.

## Discussion

**Figure 8:**
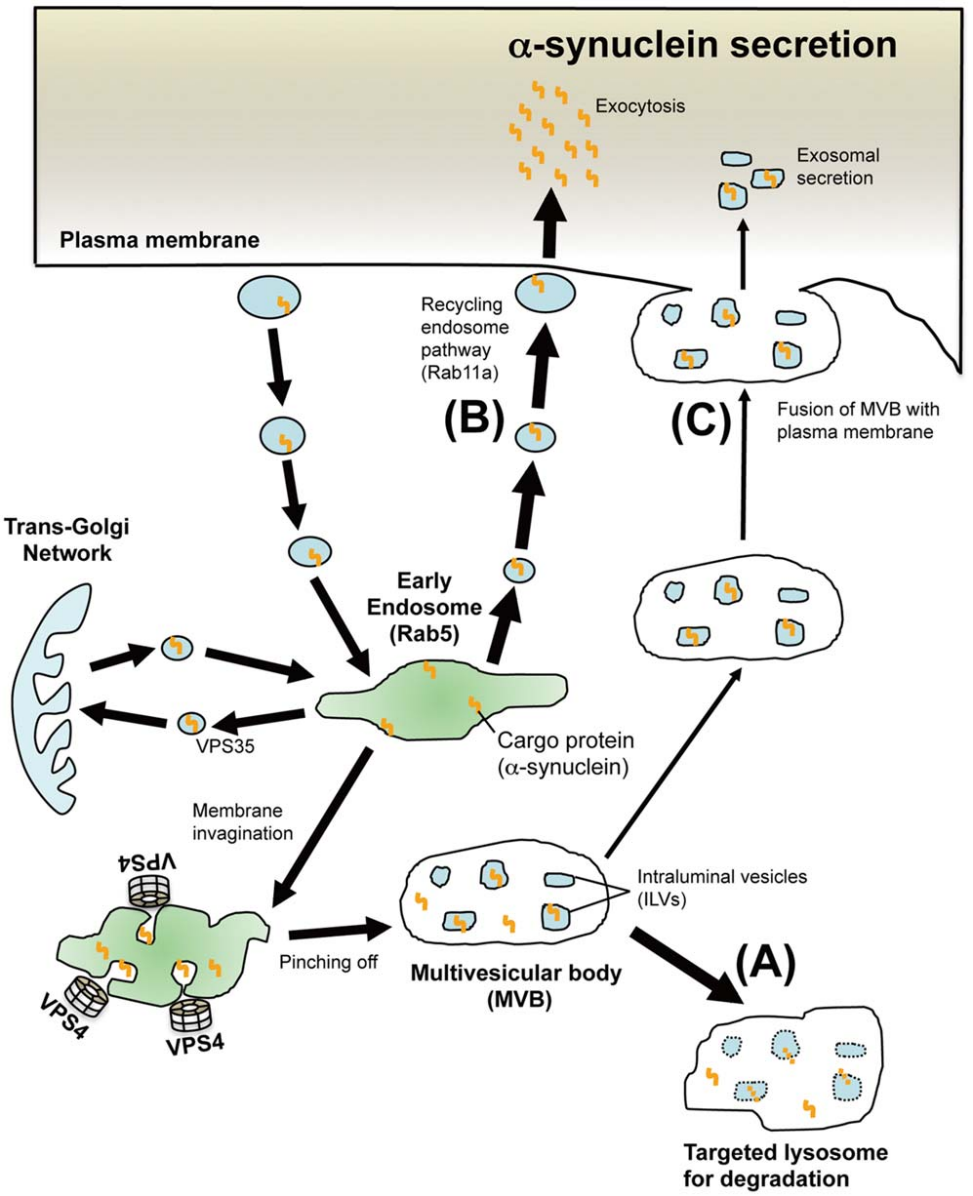
Schematic presentation of endosomal pathways and the functional relevance of MVB sorting machinery and Rab11a-mediated recycling pathway in the secretion as well as lysosomal targeting of α-synuclein. Membrane-associated cargo proteins including αSYN are translocated to early endosomes, which also receive cargoes from the trans-Golgi network. Some cargoes are recycled back to the plasma membrane. Others are sequestered in intraluminal vesicles of MVB. Individual ESCRT proteins and VPS4 contribute to MVB formation through the induction of invagination and final scission of the endosomal membrane. MVB directs either for lysosomal degradation or for secretion as exosomes by exocytic fusion with the plasma membrane. Under the physiolosical condition, αSYN in the early endosome may be transferred to MVB then targeted for lysosomal degradation (A). Alternatively, part of the endosomal αSYN may be cast into the extracellular milieu through the Rab11a-dependent recycling endosome (B) and, to a lesser degree, MVB-exosome pathway (C). If the intracellular αSYN reaches a toxic level or the MVB sorting is dammed up, excessive amounts of endocytic αSYN will flow out mainly through the recycling endosome pathway.

Until recently, αSYN has been considered to exert its physiological as well as pathogenic effects intracellularly. However, accumulating evidence suggests that both monomeric and oligomeric αSYN can be secreted into the extracellular environment, thereby affecting the normal physiological state of neighboring neuronal and glial cells [17]. In the case of prion protein, cell-to-cell transmission by means of exosome shuttle, caveolae-mediated endosomal pathway, and tunneling nanotubes has been suggested [23], [25], [52], [53]. Therefore, it is tempting to speculate that similar mechanisms could be involved in the transmission of other amyloidogenic proteins. Given that the prion enrichment and infectivity were confirmed in the cell culture media of infected cells as well as body fluids from suffering animals, prion transfer could occur by a process other than through direct cell contact [25], [41], [43]. In addition to prion protein, several reports suggested that exosomes may serve as vehicles for the transcellular spread of amyloidogenic proteins in neurodegenerative diseases including PD [17], [54], [55], [56]. As reported previously [23], [24], [25], we found a striking condensation of prion in exosomes in CM and human CSF, whereas such enrichment was not observed with αSYN (Fig. 2A, 2B and 2C). The marked discrepancy in terms of the exosomal localization implies that the secretory mechanism of αSYN might be different from that of prion protein. This idea is also supported by our findings showing that, in contrast to prion protein, the suppression of MVB-exosome biogenesis by DN VPS4A significantly increased the extracellular αSYN in non-neuronal and neuronal cells (Fig.3A and 4A). It is true that our results would seem to conflict with previous reports demonstrating that αSYN is secreted from neuronal cells by exosomes under both physiological and pathological conditions [27], [28]. However, it remains possible that αSYN might be secreted through different secretory pathways depending on the size of the aggregates or cellular condition. Indeed, part of the newly synthesized αSYN was rapidly secreted from MES cells via unconventional, endoplasmic reticulum/Golgi-independent exocytosis [49]. Another study has demonstrated that the internalized extracellular αSYN was resecreted out of neurons via a process modulated by the recycling endosome regulator Rab11a [50]. The functional importance of the recycling pathway was also verified in the cellular trafficking of amyloid-β precursor protein [57]. Our result showing that DN-Rab11a restored the aberrant αSYN secretion triggered by impaired MVB genesis also supports the functional relevance of the recycling pathway in αSYN secretion. Supposedly, under the physiological state, endosomal αSYN is destined for lysosomal degradation (Fig. 8A) or introduced into the extracellular milieu through the Rab11a-dependent recycling endosomal pathway (Fig. 8B) and, to a lesser degree, MVB-exosome pathway (Fig. 8C). However, if the intracellular αSYN reaches a toxic level or the MVB sorting pathway is dammed up for any reason, a torrent of endocytic αSYN may flow out mainly through the recycling endosome pathway. Perhaps the recycling pathway might serve as a “vent” to discharge excess αSYN that would be potentially harmful to cells. Another important finding observed in this study is that the extracellular secretion of wt-αSYN was constitutively higher than A53T mutant αSYN in mock-transfected cells as well as in DN-VPS4A engineered cells. This finding is interesting when considering the cytotoxic property of mutant αSYN, which might be liable to be entrapped inside the cells and eventually lead to cell-autonomous degeneration. It should be noted that we used cell lines over-expressing αSYN in some experiments of this study. Therefore, we cannot completely exclude the possibility that over-expressed αSYN itself might somehow affect its subcellular distribution since over-expression of αSYN hinders vesicle trafficking and recycling as a result of interaction with prenylated Rab acceptor protein 1 [58].

Since αSYN does not contain a predicted transmembrane domain or known lipid anchor, there remains a fundamental question on how it associates with endosomal vesicles. It is known that the amino-terminal amphipathic α-helical domain of αSYN is quite similar to the class A2 α-helix found in the lipid-binding motif of several apolipoproteins [59]. In fact, αSYN binds artificial liposomes containing phospholipid vesicles with acidic head groups, lipid droplets, and lipid rafts [49]. It has been shown that the portion of αSYN stably cofractionated with vesicles from brain tissues and cultured neuronal cells was not only bound to the outer membrane but certainly localized in the vesicle lumen [49]. Therefore, αSYN might be integrated into vesicles in at least two different ways. Namely, some are loosely bound to the surface of vesicles where the interaction is controlled in the balance of the free cytosolic αSYN. The others are incorporated and sequestrated into the lumen of vesicles. The mechanism by which cytosolic αSYN moves into the endosomal vesicle is poorly understood; however, apart from the vesicle permeabilization by protofibrillar αSYN [60], [61], intracellular αSYN exocytosed into the extracellular space could be internalized and directly packaged into the endosomal vesicles [15], [49], [62]. Intriguingly, it is known that the aggregation of αSYN was faster and more robust in the vesicles than in the cytosol [49], [63]. We also observed a noticeable aggregation tendency in endosomal/lysosomal αSYN and the core structures of Lewy bodies showed immnoreactivity with VPS4 Ab. These findings are interesting when considering the biogenesis of Lewy bodies, because the pale body, an early cytoplasmic change before Lewy body maturation, often contains ubiquitinated proteins as well as lysosomes and vacuolar structures [64], [65]. It is uncertain why intravesicular αSYN has a high propensity to form aggregates. However, specific environments inside the vesicle such as a high calcium concentration and low pH as well as the molecularly crowded milieu might synergistically promote αSYN fibrillization [66], [67], [68], [69]. In addition, the extensive ubiquitination of endosomal αSYN found in this study may indicate a role for ubiquitin in αSYN sorting along the endosomal pathway, since multiple monoubiquitylation and Lys-63-linked polyubiquitylation have been recognized as important sorting signals for cargo proteins in the endosome membrane [33], [70].

In summary, we found that impaired MVB-exosome biogenesis by DN VPS4A strikingly increased extracellular αSYN, which was correlated with the decreased lysosome-resident αSYN. The inhibited recycling efficiency by DN-Rab11a can not only cause a decrease of the extracellular αSYN oligomer but also restore the hypersecretion of αSYN by DN-VPS4A. Furthermore, VPS4 was found to be a component of the nigral as well as the cortical Lewy bodies. Our results demonstrate how failure of the MVB sorting machinery contributes to the extracellular secretion as well as lysosomal targeting of αSYN and may thus be involved in the propagation of Lewy pathology in PD. The importance of the endosomal/lysosomal transport system in the pathogenesis of PD is also highlighted by very recent findings that a mutation in *VPS35* gene encoding a retromer complex involved in the retrograde transport of proteins from the endosome to the trans-Golgi network causes late-onset familial PD [71], [72]. Furthermore, in a manner similar to vaccination therapy, a reduction of the extracellular αSYN brain burden by regulating the MVB sorting could be a novel therapeutic strategy for PD and other synucleinopathies. Although the concept of prion-like propagation has been recognized as a common phenomenon in many neurodegenerative diseases, it is likely that the molecular mechanisms underlying the spreading of protein-misfolding may differ depending on the biochemical nature of the protein aggregate, level of cellular stress, or the cell-type. Further studies will be needed to gain insight into the cellular mechanisms of disease progression and to identify molecular targets for therapeutic intervention in PD and other neurodegenerative diseases.

## Materials and Methods

### Plasmid Construction and Preparation

N-terminal Myc-tagged wild-type (wt) αSYN was subcloned into the *Bgl*II and *Not*I sites of pCMV mammalian expression vector (Invitrogen, Carlsbad, CA). For inducible expression, human wt and A53T mutant αSYN cDNAs were introduced into pcDNA4/TO doxycycline (Dox)-inducible expression vector (Invitrogen) using the restriction enzymes *Kpn*I and *Not*I. The plasmid pcDNA6/TR encoding tetracycline repressor protein was purchased as a part of the T-REx tetracycline-regulated mammalian expression system (Invitrogen). Triple FLAG (3xFLAG)-tagged human wt- and DN E228Q VPS4A were subcloned into the *Eco*RI and *Bam*HI sites of pCMV vector. The pEGFP-C1 plasmids encoding EGFP-tagged human wt-Rab5a, wt-Rab7, wt-Rab11a, CA-Q70L-Rab11a, DN-S25N-Rab11a were kindly provided by Dr. Mitsunori Fukuda (Laboratory of Membrane Trafficking Mechanisms, Department of Developmental Biology and Neurosciences, Tohoku University Biological Institute, Sendai, Japan). Plasmid DNAs were isolated and purified using the GenoPure Plasmid Maxi Kit (Roche, Indianapolis, IN). The fidelity and orientation of the expression constructs were confirmed by restriction enzyme digestion and/or nucleotide sequence analyses.

### Cell Culture and Transfection

HEK293T human embryonic kidney cells (kindly gifted by Dr. Taeko Miyagi, Institute of Molecular Biomembrane and Glycobiology, Tohoku Pharmaceutical University, Sendai, Japan) and SH-SY5Y human dopaminergic neuroblastoma cells (CRL-2266; American Type Culture Collection, Manassas, VA) were maintained in Dulbecco's modified Eagle's medium (DMEM; Invitrogen/GIBCO) containing 4.5g/l glucose, 2mM L-glutamine (Invitrogen) supplemented with 10% fetal bovine serum (FBS; Thermo Scientific/HyClone, Rockford, IL) at 37^o^C under humidified 5% CO_2_/air. The SH-SY5Y cell lines in which wt or A53T mutant αSYN can be induced were established using the T**-**REx expression system which consists of two key expression vectors, pcDNA4**/**TO and pcDNA6**/**TR [73], [74]. Stably transfected Dox-inducible SH-SY5Y cells were maintained in DMEM containing 4.5g/l glucose, 2mM L-glutamine supplemented with 10% FBS under selective pressure by 5 µg/ml Blasticidin and 300 µg/ml Zeocin (both from InvivoGen, San Diego, CA). HEK293T cells seeded 24 hours prior to transfection were transiently transfected using FuGENE 6 transfection reagent (Roche) at FuGENE 6 ( µl)/DNA ( µg) ratio of 3∶1. SH-SY5Y cells were nucleofected using the Nucleofector I device (LONZA AG, Cologne, Germany) with program A-023. Cells were harvested 48 hours post transfection unless otherwise stated. To evaluate αSYN decay in the presence of lysosomal inhibitor, cells were treated with bafilomycin A1 (0–10 nM dissolved in DMSO; purchased from Sigma) for 24 hours.

### Immunofluorescence Confocal Microscopy and Immunohistochemistry

Cells seeded onto UV-sterilized coverslips coated with self-made rat-tail collagen were fixed in 4% (w/v) paraformaldehyde in PBS for 10 min, permeabilized with 0.5% Triton X-100 in PBS for 5min, and blocked with 3% normal goat serum (Wako Pure Chemical Industries, Osaka, Japan) in PBS for 30min. Primary antibodies (rat monoclonal antibody (mAb) anti-DYKDDDDK (FLAG peptide)-tag (1∶500; Agilent Technologies, Foster City CA), mouse mAb anti-cMyc (clone 9E10, 1∶1000; DSHB, Iowa City, IA), rabbit pAb anti-αSYN (1∶1000, CST, Danvers, MA) and mouse mAb anti-LAMP-1 (clone H4A3, 1∶1000; DSHB)) were applied for 2 hours followed by anti-mouse IgG Alexa 488 conjugates, anti-rabbit IgG Alexa 568 conjugates, or anti-rat IgG Alexa 647 conjugates (1∶2000; Invitrogen/Molecular Probes) for 1 hour. Nuclei were counterstained with TO-PRO3 iodide and pseudo-colored as blue (Invitrogen/Molecular Probes). After immunostaining, coverslips were placed upside down on a drop of PermaFluor antifade mounting medium (Thermo Scientific). Fluorescent images were analyzed with a FV300 confocal laser scanning microscope system equipped with HeNe-Green (543 nm), HeNe-Red (633 nm) and Ar (488 nm) laser units (Olympus Corporation, Tokyo, Japan). In the multiple labeling experiments, images were collected using a single excitation for each wavelength separately and then merged using Fluoview image analyzing software (version 4.3, Olympus). For immunohistochemistry, 4-µm-thick sections of formalin fixed paraffin embedded samples including the substantia nigra and the temporal lobes from patients with PD with a mean age of 77.5 years (n = 4, range 67 to 84 years) and the controls with a mean age of 77.3 y (n = 4, range 67 to 87 years) were subjected to immunohistochemical investigations using the avidin-biotin-peroxidase complex (ABC) method with a Vectastain ABC kit (Vector Laboratories, Burlingame, CA). Polyclonal Ab against human VPS4 (SAB4200025, 1∶100; Sigma) was used as primary Ab. Diaminobenzidine was used as the chromogen. The sections were counterstained with hematoxylin. No pretreatment of sample before Ab incubation was required.

### Subcellular Fractionation by Sequential Centrifugation

For the subcellular fractionation of cultured cells, we adopted an established protocol with slight modifications [75]. All steps of the fractionation scheme were carried out at 0–4°C with ice-cold reagents. Cells (1×10^7^) were resuspended with 2 ml ice-cold fractionation buffer (10 mM Tris/acetic acid pH 7.0, 250 mM sucrose) and homogenized using 20 strokes in a 2-ml Dounce tissue grinder with a tight pestle (GPE, Bedfordshire, England). The cell homogenate was initially cleared by three successive centrifugation steps (500×*g* for 2 min, 1,000×*g* for 2 min, 2,000×*g* for 2 min) to remove debris and undestroyed cells. The supernatant was transferred to a new tube and centrifuged at 4,000×*g* for 2 min to pellet the plasma membrane and nuclei. The supernatant was ultracentrifuged at 100,000×*g* (P50S2 swing rotor, Hitachi Koki Co., Ltd., Tokyo, Japan) for 2 min to pellet the mitochondria, endosomes, and lysosomes (fraction EL). Lysosomes were isolated from the fraction EL by 10-min osmotic lysis using five times the pellet volume of distilled water. After another centrifugation step with 100,000×*g* for 2min, lysosomes remained in the supernatant, while mitochondria and endosomes were in the pellet.

### TCA/acetone Protein Extraction from Culture Medium and CSF

Total protein in CM and CSF was extracted by trichloroacetic acid (TCA)/acetone precipitation protocol. Briefly, freshly collected samples were cleared by three successive centrifugation steps (800×*g* for 5 min, 2,000×*g* for 10 min, and 10,000×*g* for 20 min at 4°C) to pellet the debris and intact cells. The supernatant was transferred to a new tube and added with an equal volume of ice-cold 20% TCA/acetone, followed by incubation at −20°C for 3 hours. After adding 3 additional volumes of ice-cold acetone, proteins were allowed to precipitate overnight at −20°C. The protein was pelleted by centrifugation at 5,000×*g* for 60min, dissolved in 8M urea/5% SDS with sonication, and subjected to Western immunoblot analyses.

### Exosome Isolation from Culture Medium and CSF

To isolate exosomes, CM or pooled CSF was collected and subjected to a multi-step differential centrifugation process. In brief, freshly collected samples were subjected to three successive centrifugations at 800×*g* for 5 min, 2,000×*g* for 10 min, and 15,000×*g* for 20 min at 4°C to remove debris and intact cells. After filtration through a 0.22 µm Millipore syringe filter, exosomes were pelleted by ultracentrifugation at 100,000×*g* (P40ST swing rotor, Hitachi Koki, Co., Ltd.) for 1 hour at 4°C. In some experiments, the exosome-containing pellet was resuspended in ice-cold PBS and further purified by continuous linear sucrose-density gradient centrifugation (2.0–0.25M sucrose, 20 mM HEPES, pH 7.2) according to the method described previously. The exosomal proteins Alix and flotillin-1 were used as markers for the exosome-containing fraction [27].

### SDS-Polyacrylamide Gel Electrophoresis and Western Immunoblot Analysis

After preparing the cell lysates using radio-immunoprecipitation assay (RIPA) buffer (1% NP-40, 0.5% deoxycholate, 0.1% sodium dodecyl sulfate (SDS), 1mM EDTA, 10mM sodium pyrophosphate, 50mM sodium fluoride, 1mM sodium orthovanadate, 150mM sodium chloride, 50mM Tris-HCl (pH 8.0) plus 1x Cømplete protease inhibitor cocktail; Roche), the protein concentration was determined using a bicinchoninic acid (BCA) protein assay kit (BioRad, Hercules, CA). Lysates containing 50 µg total protein were boiled in Laemmli loading buffer and then electrophoresed on denaturing 12.5% SDS-polyacrylamide gels using the Mini-PROTEAN 3 cell system (BioRad). Electroblotting onto polyvinylidene fluoride membrane (Immobilon-P; Millipore, Bedford, MA) was performed at 100V for 2 hours. After a blocking step with Tris-Buffer Saline (TBS: 50 mM Tris-HCl, pH 7.5, 150 mM NaCl) with 0.05% Tween 20 (TBST) supplemented with 5% nonfat dry milk, membranes were incubated with anti-cMyc mouse mAb (clone 9E10, 1∶1000; DSHB), M2 anti-FLAG/M2 (1∶1000; Sigma) mouse mAb, anti-GFP mouse mAb (1∶4000; MBL, Nagoya, Japan) anti-synuclein-1 mouse mAb (1∶1000; BD Bioscience, San Jose, CA), anti-Alix mouse mAb (clone 3A9, 1∶1000; CST), anti-flotillin-1 mouse mAb (1∶500; BD Transduction laboratories, Franklin Lakes, NJ), anti-Hsp90 mouse mAb (1∶4000; Stressgen, Victoria, BC, Canada), anti-BSA rabbit polyclonal antibody (pAb) (clone B-140, 1∶4000; Santa Cruz Biotechnology, Santa Cruz, CA), anti-prion protein mouse mAb (1∶1000; Sigma), anti-ubiquitin Ab (clone P4D1, 1∶1000; Santa Cruz), anti-LAMP-1 mouse mAb (clone H4A3, 1∶1000, DSHB), anti-Rab5 rabbit pAb (1∶4000, Santa Cruz), and anti-Rab11 rabbit pAb (1∶1000; CST, Danvers, MA). Primary antibodies were followed by horseradish peroxidase-conjugated secondary Ab (1∶10000; Jackson ImmunoResearch Laboratories, West Grove, PA). Bands were visualized with Immobilon Western Chemiluminescent HRP Substrate (Millipore) and images were captured by the LAS-3000mini lumino image analyzer (Fujifilm, Tokyo, Japan). Quantification of the band intensity was performed using the Image J version 1.44 software for Mac (developed at the National Institutes of Health, Bethesda, MD) [76]. All experiments were performed at least three times with identical results.
